# Insights into the Role of Nanorod-Shaped MnO_2_ and CeO_2_ in a Plasma Catalysis System for Methanol Oxidation

**DOI:** 10.3390/nano13061026

**Published:** 2023-03-13

**Authors:** Guangyi Zhang, Gui Chen, Haomin Huang, Yexia Qin, Mingli Fu, Xin Tu, Daiqi Ye, Junliang Wu

**Affiliations:** 1 College of Environment and Energy, South China University of Technology, Guangzhou 510006, China; 201920144886@mail.scut.edu.cn (G.Z.); 201920144862@mail.scut.edu.cn (G.C.); huanghm@scut.edu.cn (H.H.); mlfu@scut.edu.cn (M.F.); cedqye@scut.edu.cn (D.Y.); 3 Provincial Key Laboratory of Atmospheric Environment and Pollution Control, National Engineering Laboratory for VOCs Pollution Control Technology and Equipment, Guangzhou 510006, China; xin.tu@liverpool.ac.uk; 2 Department of Electrical Engineering and Electronics, University of Liverpool, Liverpool L69 3GJ, UK; qinyexia@scut.edu.cn

**Keywords:** methanol oxidation, plasma catalysis, CeO_2_, MnO_2_, in situ ft-IR

## Abstract

Published papers highlight the roles of the catalysts in plasma catalysis systems, and it is essential to provide deep insight into the mechanism of the reaction. In this work, a coaxial dielectric barrier discharge (DBD) reactor packed with γ-MnO_2_ and CeO_2_ with similar nanorod morphologies and particle sizes was used for methanol oxidation at atmospheric pressure and room temperature. The experimental results showed that both γ-MnO_2_ and CeO_2_ exhibited good performance in methanol conversion (up to 100%), but the CO_2_ selectivity of CeO_2_ (up to 59.3%) was much higher than that of γ-MnO_2_ (up to 28.6%). Catalyst characterization results indicated that CeO_2_ contained more surface-active oxygen species, adsorbed more methanol and utilized more plasma-induced active species than γ-MnO_2_. In addition, in situ Raman spectroscopy and Fourier transform infrared spectroscopy (FT-IR) were applied with a novel in situ cell to reveal the major factors affecting the catalytic performance in methanol oxidation. More reactive oxygen species (O_2_^2−^, O^2−^) from ozone decomposition were produced on CeO_2_ compared with γ-MnO_2_, and less of the intermediate product formate accumulated on the CeO_2_. The combined results showed that CeO_2_ was a more effective catalyst than γ-MnO_2_ for methanol oxidation in the plasma catalysis system.

## 1. Introduction

Volatile organic compounds (VOCs) are precursors to ozone and photochemical smog, which have negative influences on the environment and human health [1,2]. Many purification technologies have been used to remove VOCs in recent years, including adsorption, absorption, thermal catalysis, non-thermal plasma (NTP) and so on. Among these technologies, the use of NTP has been recognized as a promising air pollution purification method because it degrades low concentrations of VOCs at ambient temperature and atmospheric pressure [3]. However, at present, high-energy consumption, incomplete oxidation and low CO_2_ selectivity are the main disadvantages. The combination of NTP and catalysts (i.e., plasma catalysis) could overcome these problem [4,5]. In the plasma catalysis system, the catalysts are filled into the discharge region (in-plasma catalysis, IPC) or placed downstream of the discharge region (post-plasma catalysis, PPC). Compared with the PPC configuration, IPC induces stronger synergy between the plasma and catalyst and generally improves the conversion of VOCs and the selectivity of CO_2_ [6,7]. The role of the catalyst in the catalytic system is an important fundamental issue [8]; however, because of the complexity of the IPC system and the imperfections of in situ experimental approaches, the action mechanisms for different catalysts remain unclear to a great extent.

Transition metal oxides have the advantages of low cost, easy synthesis and high catalytic activity [9,10]. For example, CeO_2_ and MnO_2_ are both widely used in plasma catalysis and exhibit excellent performance. Among them, CeO_2_ has excellent oxygen storage/release capability and an efficient Ce^3+^/Ce^4+^ redox cycle, which provides highly active oxygen species for VOC oxidation and generally exhibits high CO_2_ selectivity [11]. For example, Zhu et al. found that CeO_2_ effectively improved the conversion to formaldehyde (65%) and especially the CO_2_ selectivity (84%) in the plasma [12]. Manganese dioxide is an excellent transition metal oxide catalyst [13,14], it can decompose ozone efficiently, which is generated in the plasma reactor by using air as the balance gas, and produces active oxygen species. A plasma catalysis system packed with MnO_2_ usually shows high conversion of VOC but low CO_2_ selectivity. Magureanu used MnO_2_-SMF in a plasma catalytic reactor for trichloroethylene oxidation and observed high trichloroethylene (95%) but low CO_2_ selectivity (32%) [15].

Investigations of the roles of the two catalysts in the plasma catalysis system, including the effect of the CeO_2_ morphology [16], the crystal structure of MnO_2_ [17] and the figure Mn/Ce ratio of the MnCeOx composites [18], have been reported widely. However, a direct and thorough comparison of these two catalysts in the plasma catalysis system has not been reported. The important features impacting plasma catalytic oxidations could be investigated by comparing the performance of these two catalysts in the process, which would serve as a guide for the design of effective catalysts appropriate for plasma catalytic oxidation.

In this work, CeO_2_ and γ-MnO_2_ were prepared via a hydrothermal method. In particular, they both showed similar features, including morphologies, sizes and specific surface areas, each of which was reported to have a significant influence on plasma catalysis [16,19,20]. Methanol was selected as the pollutant because it has a relatively simple structure, and this aids in following the oxidation pathways in the plasma catalysis process. Methanol oxidation was performed over CeO_2_ and γ-MnO_2_ in a DBD reactor. A series of experiments was designed to explore the evolution of the targeted product CO_2_, and the reactions over these two catalysts in the plasma were investigated with FT-IR spectroscopy in a novel in situ cell.

## 2. Experimental Section

### 2.1. Catalyst Preparation and Evaluation

All chemicals were analytical grade and used without further purification. In the synthesis of γ-MnO_2_, 2.7 g of MnSO_4_·H_2_O and 3.66 g of (NH_4_)S_2_O_8_ were mixed with 64 mL of deionized water and hydrothermally heated at 90 °C for 24 h. The mixture was transferred into a Teflon-lined stainless-steel autoclave with a capacity of 80 mL and heated at 160 °C for 12 h. The product was collected and washed with deionized water and finally heated at 80 °C for 12 h [21]. To prepare CeO_2_, 5 mmol of Ce(CH_3_COO)_3_·H_2_O was dissolved in 20 mL of deionized water, and then 55 mL of a 7 M NaOH solution was added to form a purple solution. The solution was transferred into a Teflon-lined stainless-steel autoclave after 0.5 h of stirring at room temperature. The autoclave was heated for 5 h at 130 °C in an oven. After cooling to room temperature, the resulting sediment was washed several times with deionized water and absolute alcohol and then dried at 100 °C for 24 h [22].

**Figure 1:**
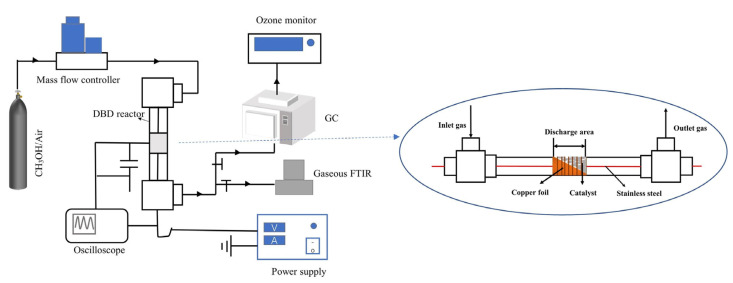
Schematic diagram of the plasma catalysis system.

Figure 1 shows a schematic diagram of the plasma catalysis system. It consisted of a gas generator, a DBD-catalyst hybrid reactor, a power supply and analysis devices. The reactant feed was 400 ppm methanol balanced with dry air, and the flow rate was 100 mL·min^−1^. The performance of plasma catalysis was determined in the DBD-catalyst hybrid reactor at 30 °C. A 6 mm long copper foil was wrapped around a 16 mm long quartz tube (o.d. = 8 mm, i.d. = 6 mm) and employed as a ground electrode, while a stainless-steel rod with a diameter of 2.0 mm was placed in the center of the quartz tube and served as the high-voltage electrode. The discharge gap between the two electrodes was 3 mm. The catalysts (0.1 g, 40–60 mesh) were placed inside the 6 mm long discharge region and fixed with quartz wool.

A high voltage alternating-current power supply (CTP-2000K, Suman Plasma Technology Co., Ltd., Nanjing, China) operating at a frequency of 1.9 kHz was used to generate the NTP. The plasma discharge characteristics were tested with an oscilloscope (DS 1052E, RIGOL Technology Co., Ltd., Beijing, China). The input power of the reactor was calculated by using the voltage-charge Lissajous method.

The outlet gas of the reactor was detected by gas chromatography (GC, Fanwei, Shanghai, China) and two FID detectors, one for detecting methanol and the other for CO and CO_2_. The latter was connected to a nickel conversion furnace, where the CO and CO_2_ were converted into methane for analysis. An ozone analyzer (2B Technology, Boulder, USA) was used to measure the concentration of O_3_. The formulas used to calculate the methanol conversion rate (X_methanol_) and reaction product selectivity S_P_ are as follows:

(1) $$X_{\mathrm{methanol}}=\frac{C_{\mathrm{methanol}}^{\mathrm{in}}-C_{\mathrm{methanol}}^{\mathrm{out}}}{C_{\mathrm{methanol}}^{\mathrm{in}}}\times 100\%$$

(2) $$S_{P}=\frac{C_{P}^{\mathrm{out}}}{\left(C_{\mathrm{methanol}}^{\mathrm{in}}-C_{\mathrm{methanol}}^{\mathrm{out}}\right)}\times 100\%$$

where P in Equations (1) and (2) represents the CO_2_ product.

### 2.2. Catalyst Characterization

The details of catalyst characterization, including powder X-ray diffraction (XRD), N_2_ adsorption, scanning electron microscopy (SEM), X-ray photoelectron spectroscopy (XPS), oxygen temperature-program desorption (O_2_-TPD), dynamic adsorption, and methanol-temperature-program desorption (methanol-TPD), are provided in the Supporting Information.

### 2.3. Investigation of the Ces for CO_2_ c

The oxygen sources for formation of the target product CO_2_ included the oxygen in the methanol molecules, active oxygen species in the catalysts, or active oxygen in the plasma (short-lived species and long-lived species). These sources have been investigated with a series of experiments (Figure S1) to clarify the role of the catalyst in the oxidation of methanol.

(1) Methanol molecules

When methanol was oxidized to CO_2_ in nitrogen plasma (Figure S1a), the oxygens contained in the methanol were transferred to give CO_2_. The concentration of methanol (balanced with N_2_) was 400 ppm, and the flow rate was 100 mL·min^−1^ (the same reaction conditions were used in the following experiments unless otherwise mentioned). The plasma was turned on with an input power of 1 W. The outlet CO_2_ was detected by gas chromatography.

(2) Active oxygen species in the catalysts

The two catalysts contained certain amounts of active oxygen species. To explore the role of these oxygen species in the plasma catalysis process, an experiment without gaseous oxygen was carried out at room temperature and with heating. The catalyst (0.1 g) was filled in the reactor, and 400 ppm of methanol was introduced. The concentrations of methanol and CO_2_ at the outlet were recorded with the GC. After adsorption saturation, the catalysts were purged with nitrogen for 1 h and then heated at a rate of 5 °C·min^−1^ from 25 °C to 450 °C. The signals of methanol and CO_2_ were recorded with the mass spectrometer (MS, Hidden HPR-20, Extratech Analytical Ltd., Beijing, China).

Then, the coupling effect of this oxygen species and the plasma was inspected. This intrinsic oxygen consumed in the oxidation of methanol was supplemented by gaseous oxygen. Multistep methanol/N_2_ discharge → oxygen → methanol/N_2_ discharge experiments (Figure S1a) were carried out to verify the ability of active oxygen species to supplement the catalyst. The discharge power was maintained at 1 W, and the concentration of CO_2_ during the process was recorded with the GC.

(3) Short-lived oxygen species

To investigate the contributions of the short-lived oxygen species (•O, •OH, O_2_*, etc.) and long-lived oxygen species (mainly O_3_), a comparison between IPC and PPC was designed. The experimental setup is shown in Figure S1b. The discharge powers of PPC and IPC were both 1 W. The catalysts in the PPC system were placed in another quartz tube 20 cm after the discharge region. Only the long-lived species reacted with the catalyst because the short-lived species were quenched immediately when leaving the discharge region. In the IPC system, the short-lived species and the long-lived species both reacted in the oxidation process. By comparing the CO_2_ selectivity of the two systems, the contributions of the short-lived species in the IPC system were estimated.

(4) Long-lived oxygen species

An ozone catalytic oxidation experiment was designed to determine the role of the long-lived species, O_3_. The experimental setup is shown in Figure S1c. High purity oxygen was passed through the discharge region at a flow rate of 10 mL·min^−1^ to generate a certain concentration of O_3_ and then mixed with methanol/N_2_ (flow rate: 90 mL·min^−1^) in a 500 mL glass bottle. After full mixing, a gas mixture containing O_3_, O_2_, N_2_ and methanol was passed into the quartz tube. The concentrations of methanol, CO_2_ and O_3_ were detected at the outlet.

### 2.4. Raman Analysis of the Reactive Oxygen Species (ROS)

In situ Raman measurements were carried out with a Raman spectrometer (Jobin Yvon Horiba, Japan) using 532 nm laser excitation. During the tests, the samples were placed in a temperature-controlled in situ cell and pretreated by passing nitrogen (100 mL·min^−1^) at 110 °C for 1 h. After cooling to 30 °C, 220 ppm of ozone was introduced to the in situ cell, and the changes in the Raman spectra of the catalyst were monitored continuously. The time intervals between data acquisitions were 15 min. After 1 h, the ozone was replaced by oxygen, and Raman signals were collected every 15 min for 2 h.

In addition, two experiments were performed to examine the ROS on the catalyst surfaces. In the first experiment, ozone and methanol were introduced simultaneously to the cell. In the second one, ozone was first introduced for 60 min, and then the gas was switched to a mixture of methanol/air.

### 2.5. Investigation of the Reaction Pathway of Methanol Oxidation

The activated species present in the discharge area were analyzed with an optical emission spectrometer (OES, ULS2048XL-EVO, AvaSpec, Beijing, China). The wavelength range was 250–1200 nm, and the full width at half maximum (FWHM) spectral resolution was 0.8 nm. The reaction pathway in the gas phase was also investigated by analyzing the exhaust gas with an infrared spectrometer (Nicolet iS5, Thermo Fisher Scientific, Waltham, MA, USA). The wavenumber range was 400–4000 cm^−1^, the resolution was 4 cm^−1^, and 32 scans were collected.

For surface reactions on catalysts, information on the intermediates and products adsorbed on the catalyst was gathered with a Fourier transform infrared spectrometer (FT-IR, Nicolet iS6700, Thermo Fisher Scientific, Waltham, MA, USA) equipped with an MCT detector (resolution: 4 cm^−1^, scan range: 740–4000 cm^−1^, scan number: 32). A novel homemade in situ cell was used for the experiments. A diagram and picture of the cell are shown in Figure S1d. The catalysts were pressed into thin slices and placed in the small slot of the inner tube between the electrodes. The discharge gap was 10.0 mm.

The catalysts were pretreated in the cell with a flow of nitrogen (100 mL·min^−1^) and then heated with infrared radiation lamps for 1.5 h to remove adsorbed water and other gas molecules. After the temperature decreased to 30 °C, a mixture of 400 ppm of CH_3_OH/air was introduced to the in situ cell until the catalysts were saturated with methanol. Finally, the gas was switched to dry air, and the power was turned on. Spectra were collected during the whole process.

In addition, some complementary in situ plasma FI-IR experiments were designed to investigate the roles of gaseous oxygen and the active oxygen species. The details of the experiments are provided in the Supporting Information.

## 3. Results and Discussion

### 3.1. Catalyst Characterization

**Figure 2:**
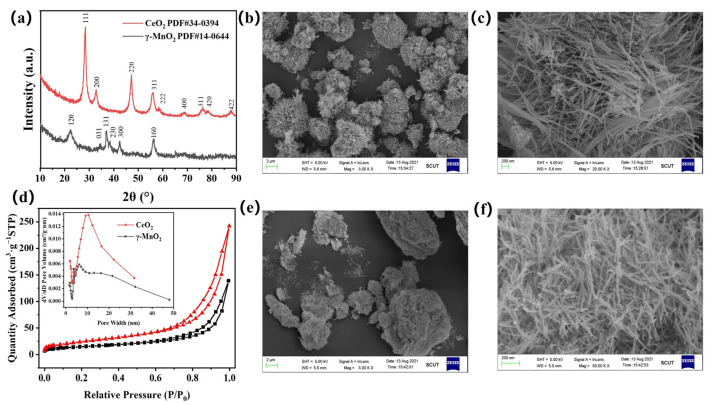
(**a**) XRD patterns of γ-MnO_2_ and CeO_2_, (**b**,**c**) SEM images of γ-MnO_2_, (**d**) N_2_ adsorption/desorption isotherms and pore size distribution curves of γ-MnO_2_ and CeO_2_, (**e**,**f**) SEM images of CeO_2_.

The XRD patterns for the two catalysts are shown in Figure 2a. The MnO_2_ exhibited the typical diffraction peaks associated with orthorhombic γ-MnO_2_ (JCPDS card No. 14-0644) [23]. The diffraction peaks of CeO_2_ at 2θ = 28.5°, 33.1°, 47.5°, 56.3° and 59.1° were assigned to the face-centered cubic fluorite structure of CeO_2_ (JCPDS card No. 34-0394) [22]. The XRD patterns of the used catalysts are shown in Figure S2. These results indicated that these two catalysts were successfully synthesized and that their structures were not damaged after the plasma catalysis reaction. The results of N_2_ adsorption measurements are shown in Table 1 and Figure 2d. Both catalysts exhibited a mesoporous morphology, and CeO_2_ had a slightly larger specific surface area and pore volume than γ-MnO_2_.

SEM images of the γ-MnO_2_ and CeO_2_ catalysts are shown in Figure 2b,c and Figure 2e,f, respectively, which show that the two catalysts both exhibited nanorod morphologies. The particle sizes of the two catalysts were similar, 1.4 μm × 180 nm for the γ-MnO_2_ nanorods and 1.2 μm × 200 nm for the CeO_2_ nanorods.

**Figure 3:**
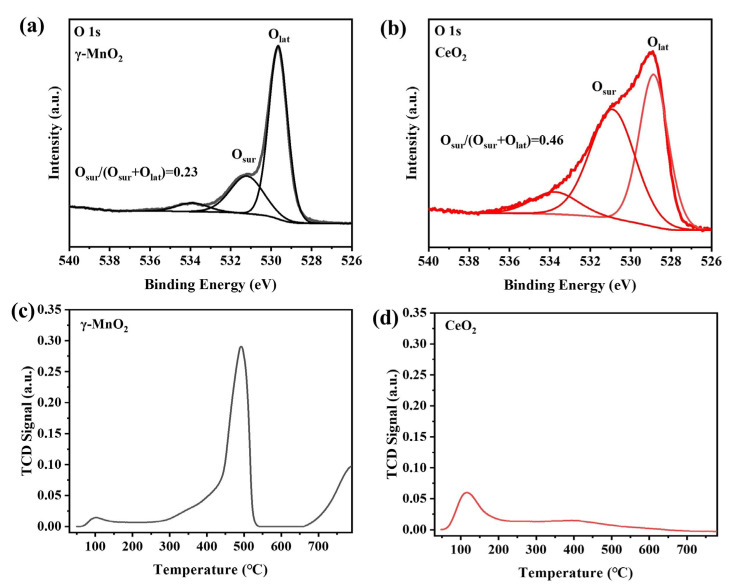
O 1s XPS spectra of (**a**) γ-MnO_2_ and (**b**) CeO_2_; O_2_-TPD spectra of (**c**) γ-MnO_2_ and (**d**) CeO_2_.

The O 1s XPS spectra of the γ-MnO_2_ and CeO_2_ catalysts are presented in Figure 3a,b. The O 1s peak was deconvoluted into two peaks corresponding to surface oxygen species (O_sur_) at 530.9 eV [24,25] and lattice oxygen species (O_lat_) [24] at 528.9 eV. The ratio O_sur_/(O_sur_ + O_lat_) reflected the contents of the active oxygen species in the catalysts. The values are shown in Figure 3a,b. The O_sur_/(O_sur_ + O_lat_) ratio of CeO_2_ was 0.46, which was twice as high as that of γ-MnO_2_, indicating that the CeO_2_ sample contained more surface oxygen species than the γ-MnO_2_ sample. Considering that active oxygen species (such as O_2_^2−^ and O^2−^) are highly reactive, it could be inferred that CeO_2_ may perform better in the oxidation of methanol.

O_2_-TPD was used to investigate the interactions and activation of oxygen molecules on the catalyst surface, and the results are shown in Figure 3c,d. Three oxygen desorption peaks were observed between 50 °C and 800 °C for the γ-MnO_2_ sample. The oxygen desorption peak at 103 °C was attributed to physically adsorbed oxygen, and the desorption peaks at 493 °C and 789 °C were ascribed to subsurface lattice oxygen and bulk lattice oxygen, respectively [26]. Two oxygen desorption peaks were presented for CeO_2_ in the same temperature range. The peak at 112 °C was attributed to the desorption of physically adsorbed oxygen. The peak at ~400 °C was ascribed to absorbed superoxide (O_2_^−^) and peroxide (O^−^) species [11]. A comparison of these two samples showed that more active oxygen species were desorbed from the CeO_2_ surface at low temperature than from γ-MnO_2_ (<200 °C). This was consistent with the XPS results.

### 3.2. Plasma Catalysis Performance Evaluation

**Figure 4:**
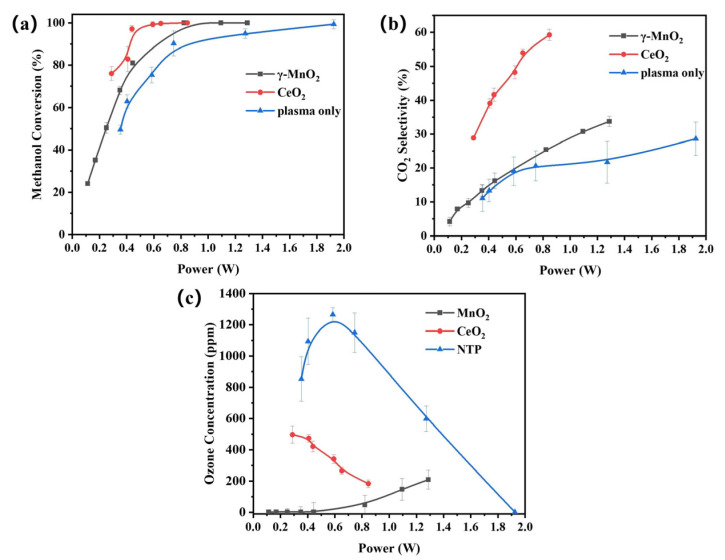
Degradation of methanol in the plasma catalytic and plasma-only systems: (**a**) methanol conversion, (**b**) CO_2_ selectivity, and (**c**) outlet ozone concentration (methanol concentration: 400 ppm, catalyst amount: 0.1 g, gas flow rate: 100 mL·min^−1^, temperature: 30 °C).

The activities of γ-MnO_2_ and CeO_2_ for methanol oxidation in the plasma catalysis systems were evaluated, and the results are shown in Figure 4. The methanol conversion increased with increasing input power; however, the power required for complete conversion differed for the two catalysts and the plasma alone. CeO_2_ exhibited the highest methanol conversion rate at low input power. The power required for complete conversion increased in the order CeO_2_ (0.9 W) < MnO_2_ (1.3 W) < plasma only (1.8 W).

Moreover, as shown in Figure 4b, CeO_2_ exhibited much higher CO_2_ selectivity than γ-MnO_2_ and plasma only. It reached 59.8% at a power of 1 W, whereas those of γ-MnO_2_ and plasma-only reached 23% and 20%, respectively. The plasma-only system showed the worst performance.

In the plasma-only system, methanol oxidation mainly depended on collisions between the methanol molecules and active species produced by filamentary discharge. The CO_2_ selectivity of the plasma-only system was fairly low, and it generated more byproducts and exhibited lower energy efficiency. After introduction of the two catalysts, the performance was improved, and the surface catalytic reactions of the catalysts played positive roles in methanol oxidation. CeO_2_ exhibited better performance than γ-MnO_2_, which may have resulted because it had more surface oxygen species, as shown in the XPS and O_2_-TPD spectra. These surface oxygen species could be utilized effectively in the surface reactions in the plasma, which would enhance the oxidation of methanol. The dominant contribution should be ascribed to the interactions between the catalysts and the active species in the plasma.

Ozone, an inevitable byproduct in a plasma catalysis system when using air as the balance gas, should be eliminated in the tail gas. As shown in Figure 4c, the emission ozone concentration of γ-MnO_2_ was lower than that of CeO_2_, suggesting that γ-MnO_2_ decomposed more ozone than CeO_2_. Generally, catalysts with better ozone decomposition capability perform better in the plasma catalysis systems used for VOC oxidation. The catalysts decompose ozone into ROS, such as O, O^−^, O^2−^ and O_2_^2−^ [27]. ROS exhibit better performance than ozone for VOC oxidation [28,29]. However, the methanol conversion and CO_2_ selectivity of γ-MnO_2_ were lower than those of CeO_2,_ which seems to contradict previous studies. The results of these experiments are discussed in a later section.

## 4. Mechanistic Studies on the Roles of the Catalysts

### 4.1. Electrical Discharge Characteristics

Figure S3a–c shows the Lissajous curves for the plasma-only and plasma catalytic systems with γ-MnO_2_ and CeO_2_ at an input power of 1 W. The Lissajous curves for the plasma-only system (Figure S3a) and the system containing CeO_2_ (Figure S3c) were similar but differed from the system with γ-MnO_2_ (Figure S3b). Figure S3d–f shows the voltage and current waveforms of the three systems. The current waveforms in Figure S3d,f indicated that the plasma-only system and the system with CeO_2_ both exhibited typical filamentary discharge with evident oscillations, which is an unstable microdischarge state. In contrast, Figure S3e shows that the oscillations of the current waveforms became weaker in the case of γ-MnO_2_, which was a sign of surface discharge. The dielectric constants of γ-MnO_2_ and CeO_2_ are approximately 8000 and 10 [30,31] depending on the temperature, pressure, power frequency, material shape and thickness of the material. Normally, γ-MnO_2_ has a larger dielectric constant, and when the DBD reactor is filled with γ-MnO_2_, gap discharge is easily converted to localized discharge [32]. This is why the current waveforms of γ-MnO_2_ consisted of many smaller oscillations during each half cycle. The dielectric constant affected the electric field in the voids and therefore altered the mean electron energy, which affected the distribution of active species in the plasma. The specific influence will be shown in the OES spectra.

### 4.2. Active Species in the Plasma

**Figure 5:**
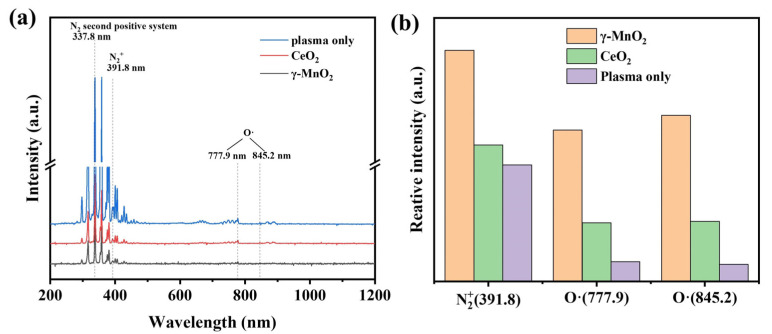
(**a**) OES spectra for plasma only and with γ-MnO_2_ and CeO_2_ and (**b**) a comparison of the normalized peak intensities (methanol concentration: 400 ppm, balance gas: air, gas flow rate: 100 mL·min^−1^, 1.9 kHz, 1.5 W).

OES analysis was used to detect the excited and ionized species in the plasma, and the results are shown in Figure 5a. The appearance of the N_2_^+^ peak (B^2^Σu^+^ → X^2^Π_g_^+^) indicated that nitrogen ionization (e + N_2_ → e + N_2_^+^ + e) occurred during the reaction [33,34], and its intensity was related to the number of high-energy electrons. The excited state of oxygen (denoted as O·) was produced by the excited-state dissociation of oxygen in the electric field [35]. As seen from Figure 5a, the peak intensity was highest for the plasma alone and decreased for the catalyst because the catalyst blocked the propagation of light and resulted in a decrease in the overall intensity. To exclude systematic errors and temperature changes occurring during the test, all three spectra signals were normalized according to the strongest nitrogen peak at 337.8 nm. The normalization process is described in the Supporting Information. The relative intensities of the peaks for N_2_^+^ (391.8 nm) and O·(777.9 nm, 845.2 nm) are shown in Figure 5b. They were highest for γ-MnO_2_, followed by CeO_2_ and the plasma-only system. This indicated that addition of the catalyst increased the local field strength and generated more plasma species. γ-MnO_2_ has a larger dielectric constant than CeO_2_ and can increase the local field strength, therefore generating more plasma species. However, on the other hand, the stronger oxygen peak for γ-MnO_2_ also indicated that this catalyst could not effectively utilize the active oxygen-containing species, i.e., more active oxygen species were generated and diffused into the discharge area without colliding with the surface of the catalyst. CeO_2_ reacted with more plasma-induced active species, which enhanced the catalytic oxidation of methanol [33].

### 4.3. Methanol Adsorption and Activation on the Catalysts

In the plasma catalytic system, the absorption and activation abilities of the catalysts greatly affect their catalytic performance. Dynamic adsorption and methanol-TPD-MS were used to investigate methanol adsorption on the two catalysts. In addition, the evolution of CO_2_ was related to the activation ability of the catalysts.

**Figure 6:**
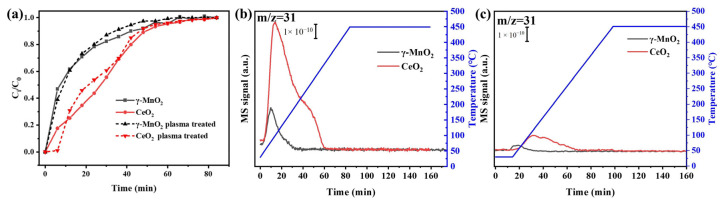
(**a**) Dynamic adsorption curves for fresh and used catalysts (methanol concentration: 400 ppm, catalyst amount: 0.1 g, gas flow rate: 100 mL·min^−1^, temperature: 30 °C); methanol-TPD-MS profiles of the catalysts after absorption (**b**) without plasma and (**c**) with plasma (power: 1 W).

Figure 6a shows the dynamic adsorption curve of methanol on the two catalysts. The adsorption curve is calculated using the equation S1. The methanol adsorption capacity of CeO_2_ was higher than that of γ-MnO_2_, which were 0.0080 g·g^−1^ for CeO_2_ and 0.0015 g·g^−1^ for γ-MnO_2_. Furthermore, the treatment with air plasma did not substantially change the adsorption capacities of the two catalysts, which were 0.086 g·g^−1^ for CeO_2_ and 0.016 g·g^−1^ for γ-MnO_2_. The specific surface area of CeO_2_ was larger than that of γ-MnO_2_; however, the difference in adsorption capacity is not proportional to that of the specific surface area. This meant that chemical adsorption of methanol occurred on CeO_2_.

The chemical adsorption of methanol on the two catalysts was explored using methanol-TPD-MS. The MS signal of methanol in the desorption process is shown in Figure 6b. The peak area of methanol desorption on CeO_2_ was significantly larger than that of γ-MnO_2_. More methanol was chemically adsorbed on CeO_2_ than on γ-MnO_2_, which should improve the oxidation of methanol in the plasma.

In particular, methanol adsorption in the plasma was investigated. Figure 6c shows the desorption curves for methanol after the catalysts were saturated with methanol in the discharge region. In comparison with Figure 6b, Figure 6c shows that the areas of the methanol desorption peaks of the two catalysts were significantly decreased. This meant that the active species in the plasma consistently decreased methanol adsorption consistently on the surfaces of the catalysts. However, the peak area of methanol desorption of CeO_2_ was still larger than that of γ-MnO_2_, suggesting a stronger interaction between methanol and the surface of CeO_2_ relative to that for γ-MnO_2_, even in the plasma.

### 4.4. Oxygen Species for CO_2_ Formation

The O elements in the product CO_2_ may have been derived from methanol, active oxygen species in the catalysts, or active oxygen species produced in the discharge area, which included short-lived species and long-lived species. A series of experiments was designed to distinguish the contributions of the oxygen species mentioned above.

The nitrogen plasma experiment (Section 2.3 (1)) was performed without a catalyst to explore the source of oxygen from the methanol molecules. As shown in Figure S4a, the low concentration of CO_2_ indicated that this oxygen source was modest.

In addition, the active oxygen species in the catalysts were investigated. Figure S4b shows the amount of CO_2_ generated by the catalysts in a flow of methanol/N_2_ at room temperature. A small quantity of CO_2_ was detected, indicating that the active oxygen species in the catalysts oxidized methanol at room temperature; however, this oxygen was consumed without replenishment, and the production of CO_2_ decreased gradually. This meant that the contributions of active oxygen species in the catalysts were insignificant and unsustainable at room temperature. When the temperature was increased or the plasma was introduced, the active oxygen species in the catalysts became more active and participated in the catalytic oxidation of methanol. The effect of temperature was also investigated here. The methanol-saturated catalysts were heated in a flow of N_2_, and the CO_2_−TPD−MS profiles are shown in Figure S4c. The absorbed methanol was oxidized by the active oxygen species in the catalysts at elevated temperatures.

To explore whether the active oxygen species in the catalysts were activated by the plasma, an experiment consisting of methanol/N_2_ discharge → oxygen → methanol/N_2_ discharge was performed, and the results are shown in Figure S5. This showed that γ-MnO_2_ produced approximately 120 ppm of CO_2_ when the nitrogen plasma was turned on. Then, the CO_2_ concentration began to decrease rapidly after 36 min and stabilized at a certain concentration. The oxygen source of CO_2_ at this stage was the methanol molecules themselves. After 30 min of oxygen purging, nitrogen was introduced, and then the plasma was turned on again. At this time, the production of CO_2_ could not be restored to the concentration seen in the first 30 min, suggesting that the active oxygen species in γ-MnO_2_ were activated by the plasma and participated in the oxidation of methanol but were not supplemented by the gaseous oxygen after consumption. In contrast, Figure S5b shows that methanol decomposition on CeO_2_ in the nitrogen plasma produced 180 ppm of CO_2_ when the plasma was turned on, and then the concentration rapidly decreased and stabilized. Then, after the same oxygen purging process, the plasma was turned on again, and the level of CO_2_ production on CeO_2_ reached a higher concentration instantaneously and then decreased gradually, indicating that the active oxygen species in CeO_2_ that participated in the methanol reaction in the nitrogen plasma were supplemented by gaseous oxygen after consumption and then participated in the reaction.

**Figure 7:**
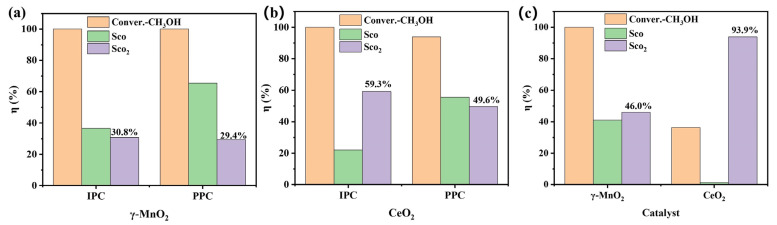
Comparison of γ-MnO_2_ and CeO_2_ in the IPC and PPC systems and ozone catalytic oxidation for methanol oxidation: (**a**) IPC and PPC with γ-MnO_2_ and (**b**) IPC and PPC with CeO_2_ (methanol concentration: 400 ppm, balance gas: N_2_, gas flow rate: 100 mL·min^−1^, temperature: 25 °C, power: 1 W); (**c**) ozone catalytic oxidation (methanol concentration: 400 ppm, ozone concentration: 900 ppm, gas flow rate: 100 mL·min^−1^, temperature: 30 °C).

Figure 7ab shows a comparison of the IPC and PPC systems with catalysts for methanol oxidation. The CO selectivities of both catalysts in IPC was less than that in PPC, indicating that the interactions between the plasma and the catalysts lowered the production of CO. The CO_2_ selectivity in IPC by CeO_2_ was 10% higher than that in PPC, whereas the CO_2_ selectivity of γ-MnO_2_ were similar in the two systems. In general, the IPC system exhibited better performance than the PPC system, which may be due to their different configurations. IPC had the combined contributions of the reaction between the long- and short-lived species and the catalysts, whereas PPC had no contribution from the reaction between the short-lived species and the catalysts. Therefore, the suppression of CO formation and enhancement of the CO_2_ selectivity with IPC indicated that the contributions of the short-lived species were important, which was more significant with CeO_2_.

Figure 7c shows the results for ozone-assisted catalysis of methanol oxidation. The CO_2_ selectivities of CeO_2_ and γ-MnO_2_ were 46% and 94%, respectively. This indicated that the effects of the long-lived species, mainly O_3_, on the CO_2_ selectivity were much greater than those of the active oxygen species in the catalyst and the short-lived oxygen species.

It is speculated that the CO_2_ selectivity of ozone catalytic oxidation of methanol was higher than that of the IPC and PPC systems due to two main reasons. First, in the IPC system, the plasma may have inhibited the chemical adsorption of methanol on the catalyst. The methanol TPD-MS spectra in Figure 6c show that, after the adsorption of methanol in the plasma, the methanol desorption peak was much smaller than that seen without the plasma. During bombardment by the plasma, some methanol molecules were desorbed into the gas phase, which inhibited the deep oxidation of methanol. Second, in the PPC system, the methanol first passed through the discharge area. This process produced many byproducts, which were not completely oxidized by the catalyst in the second reactor. However, in the ozone-catalytic experiment, the ozone and methanol reacted directly on the catalyst, which enabled direct utilization of the active oxygen species generated by ozone degradation and promoted the deep oxidation of methanol.

The above analysis showed that O_3_ was the main source of oxygen in the complete decomposition of methanol to CO_2_ and H_2_O in the plasma catalytic process. However, O_3_ alone was not active enough to complete this process, and various ROS generated from O_3_ decomposition played important roles in the deep oxidation of methanol. It is necessary to analyze the process of O_3_ decomposition over the catalysts further.

### 4.5. In situ Raman Analysis of ROS

**Figure 8:**
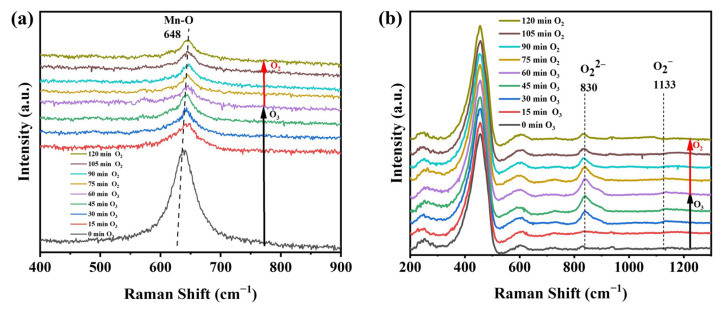
Raman spectra for O_3_ decomposition on (**a**) γ-MnO_2_ and (**b**) CeO_2_.

The decomposition of O_3_ over the two catalysts was observed with in situ Raman spectroscopy. Raman spectra of the two catalysts were obtained after passing O_3_ for 1 h and then O_2_ for 1 h, and these are shown in Figure 8. No peaks of oxygen species were observed during the whole process with γ-MnO_2_ (Figure 8a). The Raman peak at 648 cm^−1^, which was ascribed to the vibration of a Mn-O bond, decreased gradually with the introduction of O_3_ [36], and no recovery was observed with flowing O_2_. It is speculated that the structure of γ-MnO_2_ was involved in the decomposition of O_3_, which changed the lengths of the Mn-O bonds and led to a decrease in the frequency of the Raman peak at 648 cm^−1^. Figure 8b shows the Raman spectra of CeO_2_. Within the first 1 h under flowing O_3_, the peak at 830 cm^−1^ for O_2_^2−^ showed significant increases with time, and a small and flat peak for O^2−^ was also observed at 1133 cm^−1^ [37]. This indicated that the O_3_ decomposed on the surface of CeO_2_ to generate ROS, such as superoxide or peroxide species. When passing O_2_ for 1 h, the intensity of the peak at 830 cm^−1^ gradually decreased, and the peak at 1133 cm^−1^ disappeared, suggesting a decrease in the active oxygen species accumulated on the CeO_2_ surface.

Ozone and the decomposed ROS were the key active species promoting deep oxidation of methanol on the catalyst surface [27,38]. CeO_2_ decomposed the O_3_ into the reactive oxygen species, and these species completely oxidized methanol and its byproducts (formaldehyde, formic acid, methyl formate, etc.) adsorbed onto the catalyst surface into CO_2_ and H_2_O. As CeO_2_ absorbed more methanol than γ-MnO_2_, these reactive oxygen species reacted more efficiently with the absorbed methanol and ultimately increased the conversion of methanol and the selectivity for CO_2_. In contrast, less methanol was adsorbed on γ-MnO_2_, and fewer oxygen species were generated after O_3_ decomposition, most of which self-decomposed and were unable to react with the adsorbed methanol.

## 5. Reaction Pathway

### 5.1. Reactions in the Gas Phase

Figure S6 shows the infrared spectra of the outlet gas from the plasma-only and plasma catalytic systems. These showed that, without a catalyst, the methanol was decomposed by the plasma and produced methoxy, formaldehyde, formate, O_3_, carbonate, CO, CO_2_, methyl formate and some nitrogen-containing byproducts (N_2_O). Strong peaks were observed for O_3_ at 1016 cm^−1^ and 2124 cm^−1^ [39]. In the presence of the catalyst, the intensities of the peaks for O_3_ were significantly decreased, indicating decomposition of the O_3_ on the catalysts. Moreover, the peaks at 1672 cm^−1^ and 1300 cm^−1^ for formate decreased [40], and small peaks emerged in the range 1300–1600 cm^−1^, which were attributed to carbonate species [41]. The peaks for CO_2_ and CO appeared at 2359 cm^−1^ and 2165 cm^−1^, respectively [42]. In addition, a peak for a nitrogen-containing byproduct, perhaps N_2_O, was observed at 2236 cm^−1^. Based on the above analysis, a reaction pathway for methanol is proposed. Methanol was oxidized to methoxy, formate and formaldehyde in the discharge area; then, the catalyst decomposed O_3_ to produce ROS, and the methoxy, formate and carbonates were further oxidized by the ROS to CO and CO_2_. The difference between the two catalysts was that the plasma catalytic system with γ-MnO_2_ produced more methyl formate (1765 cm^−1^) [31], and the peak for CO_2_ was obviously weaker than that produced by CeO_2_. This showed that the plasma oxidation of methanol catalyzed by γ-MnO_2_ produced more byproducts, and the mineralization degree was lower than that of CeO_2_.

### 5.2. Reactions on the Surfaces of the Catalysts

**Figure 9:**
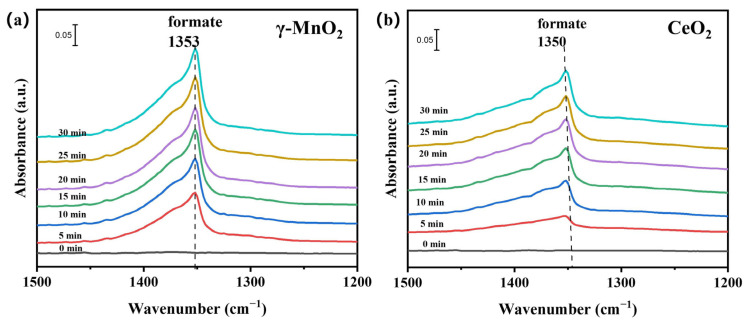
(**a**) FT-IR spectra of the plasma area during the reaction (methanol concentration: 400 ppm, balance gas: air, gas flow rate: 100 mL·min^−1^, 1.9 kHz, 0.6 W); (**b**) in situ FT-IR spectra of the surfaces of the catalysts (air flow rate: 10 mL·min^−1^, 1.9 kHz, 0.6 W).

As mentioned above, the oxygen sources of CO_2_ formation were methanol, the catalyst itself and the short-lived and long-lived oxygen species in the gas phase. After pretreatment, the catalysts were purged with methanol/air until they reached saturation. Then, the mixture of methanol/air was replaced with dry air, and the plasma was turned on. Figure 9ab shows the IR spectra of the catalyst surfaces obtained in the air plasma, and there were different amounts of formate accumulated on the surface, as indicated by the bands at ~1353 cm^−1^ for symmetric vibrations and at 1600 cm^−1^ for asymmetric vibrations. It has been reported that when the frequency difference between these two vibrational modes (1353 and 1600 cm^−1^) is less than 250 cm^−1^, the formate adsorbed on the catalyst is bound as monodentate formate [40]. These two catalysts presented the same absorption mode for formate, but the γ-MnO_2_ showed significant accumulation of monodentate formate. The intensity of the peak at 1353 cm^−1^ increased gradually with time and was much stronger than that of CeO_2_. The peak at 1600 cm^−1^ disappeared from the CeO_2_ spectrum, which indicated that the formate was gradually consumed on CeO_2_. This also explains why the selectivity of γ-MnO_2_ was worse than that of CeO_2_. Regarding how oxygen and nitrogen in the atmosphere, as well as active oxygen species of the catalyst, play a role in the catalytic process, further experiments were conducted in the Support Information, as shown in Figures S7–S10 [40,43,44].

### 5.3. Deduction of the Reaction Pathway

**Figure 10:**
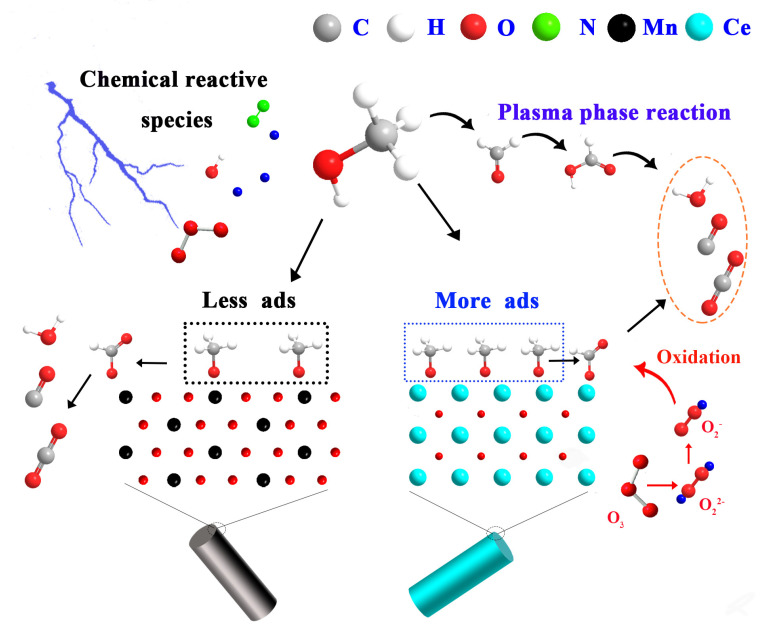
Plausible reaction pathways for methanol oxidation in the plasma catalysis system.

Based on the above analyses, mechanisms are proposed in Figure 10 for methanol oxidation in the plasma catalytic system. In the gas phase, the methanol is decomposed into formaldehyde, formic acid and methyl formate by the plasma, and then these intermediates are further decomposed into CO, CO_2_ and H_2_O. After methanol was adsorbed on the surface of the catalyst, methoxy was formed and then further oxidized to formate. During the plasma discharge, the high-energy electrons dissociated the oxygen molecules and formed ozone molecules through collisions. Then ozone was decomposed to form active oxygen species on the catalyst surface. These active oxygen species reacted with intermediates such as methoxy and formate and improved the CO_2_ selectivity.

## 6. Conclusions

In summary, the coupled effects of the γ-MnO_2_ or CeO_2_ catalysts with a nonthermal plasma used for methanol decomposition were investigated in this work. The results showed that the CeO_2_ nanorods exhibited better performance than the γ-MnO_2_ nanorods, especially for the deep oxidation of methanol. CeO_2_ adsorbed and activated more methanol in the plasma catalysis system than γ-MnO_2_. Compared with γ-MnO_2_, the CeO_2_ contained more active oxygen, utilized more oxygen-plasma species and ozone and then produced ROS in the plasma catalysis system, which were beneficial for the deep oxidation of methanol. In addition, the FT-IR experiments on the in situ plasma showed that methanol was decomposed both in the gas phase and on the surfaces of the catalysts. Different oxygen species participated in these processes with varying contributions to the deep oxidation of methanol.

## Figures and Tables

**Table 1:** Results of N_2_ adsorption of γ-MnO_2_ and CeO_2_.

Sample	Specific Surface Area/(m²·g^−1^)	Total Pore Volume/(cm^3^·g^−1^)	Average Pore Size/(nm)
γ-MnO_2_	61.43	0.15	15.83
CeO_2_	90.49	0.28	12.34

## Data Availability

The data are available from the corresponding author on reasonable request.

## Supplementary Materials

The following supporting information can be downloaded at: https://www.mdpi.com/article/10.3390/nano13061026/s1, Figure S1: Schematic diagram of (a) investigation of the methanol molecules and catalyst’s in-trinsic oxygen, (b) the comparison of IPC and PPC, (c) ozone catalytic oxidation, (d) in situ plasma catalysis FT-IR cell; Figure S2: XRD pattern of used γ-MnO2 and CeO2; Figure S3: Lissajous curves for the (a) plasma-only system, plasma catalysis system with (b) γ-MnO2, and (c) CeO2; voltage and current waveforms for the (d) plasma-only system, plasma catalysis system with (e) γ-MnO2 and (f) CeO2 at an input power of 1 W; Figure S4: (a) CO2 production in methanol plasma-catalytic experiment without catalysts (b) CO2 production in catalytic experiments at room temperature without oxygen in the gas phase; (c) CO2-TPD-MS profiles for catalysts after the catalytic experiments at room temperature (methanol concentration: 400 ppm, balance gas: N2, gas flow rate: 100 mL·min^−1^); Figure S5: Evolution of outlet CO2 of the plasma catalyst systems with different atmosphere (methanol concentration: 400 ppm, balance gas: N2, catalyst amount: 0.1 g, gas flow rate: 100 mL·min^−1^, temperature: 30 °C, power: 1 W); Figure S6: The FT-IR spectra of the plasma area during the reaction (methanol concentration: 400 ppm, balance gas: air, gas flow rate: 100 mL·min^−1^, 1.9 kHz, 0.6 W); Figure S7: In situ FT-IR spectrum of methanol adsorption for (a) γ-MnO2 and (b) CeO2 at room temperature (methanol concentration: 400 ppm, balance gas: air, gas flow rate: 100 mL·min^−1^); Figure S8: In situ FT-IR spectrum of N2 plasma catalytic oxidation for (a) γ-MnO2 and (b) CeO2 after the adsorption at room temperature (nitrogen flow rate: 10 mL·min^−1^, 1.9 kHz, 0.9 W); Figure S9: In situ FT-IR spectra of methanol degradation by catalyst own oxygen species combined with airnitrogen plasma for (a) γ-MnO2 and (b) CeO2 (N2 flow rate: 10 mL·min^−1^, 1.9 kHz, 0.6 W): Figure S10: In situ FT-IR spectra of methanol degradation by catalyst own oxygen species combined with nitrogen plasma for (a) γ-MnO2 and (b) CeO2 (N2 flow rate: 10 mL·min^−1^, 1.9 kHz, 0.6 W).
