# Supplementary material for: Insights into the Role of Nanorod-Shaped MnO2 and CeO2 in a Plasma Catalysis System for Methanol Oxidation
Source: Nanomaterials (Basel). 2023 Mar 13;13(6):1026. doi: 10.3390/nano13061026 (PMC10057529; doi:10.3390/nano13061026)
Supplement: Supplementary file 1 [file nanomaterials-13-01026-s001.zip › nanomaterials-2232874-supplementary.pdf]

nanomaterials

Supporting Information

Details of Experiments

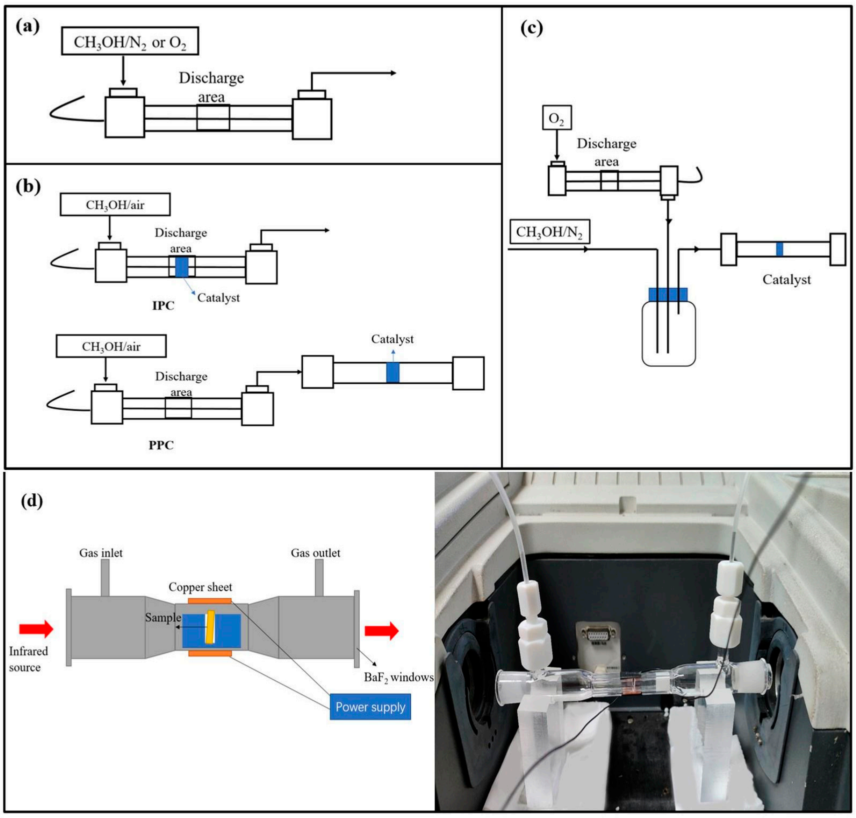

Figure S1. Schematic diagram of (a) investigation of the methanol molecules and catalyst's intrinsic oxygen, (b) the comparison of IPC and PPC, (c) ozone catalytic oxidation, (d) *in situ* plasma catalysis FT-IR cell.

Catalyst characterization

Powder X-ray diffraction (XRD) patterns were collected with Cu Kα radiation (40 kV, 40 mA, scanning step = 0.02°) at a scanning rate of 4 °·min^{-1} in the 2θ range of 5–90° on a D8 ADVANCE diffractometer (Bruker, Germany). The Brunauer–Emmett–Teller (BET) specific surface area was measured using a Micromeritics ASAP 2020 apparatus after vacuum degassing with 150°C for 5 h. The morphology of catalysts was analyzed by SEM (MERLIN, Carl Zeiss, Germany). The catalysts were also analyzed by ESCALAB 250Xi (Thermo SCIENTIFIC, USA) X-ray photoelectron spectroscopy (XPS) with Al Kα (hν=1486.8 eV) as the excitation source. O_2-TPD, as well as methanol-TPD, were carried out on a chemisorption analyzer (Micromeritics AutoChem II2920, USA) equipped with a thermal conductivity detector (TCD).

Dynamic adsorption experiments were used to evaluate methanol adsorption capacity. The inlet methanol concentration was controlled at 400 ppm and balanced with dry air to keep the gas flow at 100 mL·min^{-1}. The steam entered into a fixed bed in the quartz column containing 0.1 g of γ-MnO_2 and CeO_2 respectively as the adsorbent. The inlet and outlet concentrations of methanol were determined by gas chromatography. The adsorption capacity was calculated using the integral of the breakthrough curves, as shown in equation. S1.

$$q = \frac{FC_0 10^{-9}}{m} \left[t_s - \int_0^{t_s} \frac{C}{C_0} dt\right] \tag{S1}$$

where q is the estimated maximum adsorption capacity ($g \cdot g^{-1}$), F ($mL \cdot min^{-1}$) is the total gas flow, C_0 and C are the inlet and outlet methanol concentrations ($mg \cdot m^{-3}$), respectively, m is the mass of adsorbent (g), and t_s is the adsorption time (min).

Furthermore, to investigate the effect of plasma on methanol adsorption, the catalyst was first treated with air plasma (0.65 W), and then 0.1 g of the treated catalyst was carried out in the same dynamic adsorption experiments.

In addition, methanol chemical adsorption of the catalysts was inspected by a mass spectrometer (MS). After adsorption of methanol reached saturation, 0.1 g catalyst was purged with high purity nitrogen for 1 h until the baseline was stable. The saturated catalyst was heated by a temperature-programmed controller, and the heating rate was increased from 30°C to 450°C at a rate of 5°C·min^{-1}. The signals of methanol during the heating process were recorded by an MS. To explore the effect of plasma on adsorption characteristics of the catalyst, the previous adsorption process at room temperature was changed to perform coupling with plasma (the power was 0.65 W), that is to say, methanol was absorbed on the surface of the catalyst when the plasma is turned on until the saturation. After the saturated catalyst was purged with high purity nitrogen for 1 h, it was heated with the same heating program to record the MS signal of methanol.

Calculation of the normalized relative intensity of OES

To compare the relative intensities of species (N_2^+, O·) identified in a given OES spectrum, their values were normalized initially against the (0, 0) band head of N_2 second positive system in the spectrum, which was assigned the value 1.

Complementary *in situ* plasma FI-IR experiments

The first experiment was to explore the role of gaseous oxygen in the plasma catalytic system. The catalyst was pretreated by heating and nitrogen purging, and then methanol/N_2 was introduced. After the catalyst was adsorbed and saturated with methanol, high-purity nitrogen was introduced and purged the methanol, and then the plasma was turned on. After a period of discharge, the high-purity nitrogen was replaced with dry air (containing 21% oxygen) and the plasma is maintained. The adsorption and discharge in nitrogen consumed intrinsic oxygen of catalysts. In the air plasma, the majority source of oxygen was from the gas phase, the contribution of gaseous oxygen can be observed.

The second experiment was to explore the role of catalyst's intrinsic oxygen. Methanol/N_2 was injected after the catalyst was pretreated by heating. After the catalyst was saturated with methanol, the high-purity oxygen was introduced for 0.5 h. Because the adsorption would consume part of the catalyst's intrinsic oxygen, and the purpose of introducing high-purity oxygen was to complement the catalyst's intrinsic oxygen. Then, the high-purity O_2 was replaced by high-purity N_2, and the plasma was turned on. At the moment, the only oxygen source is the catalyst's intrinsic oxygen supplemented by O_2, so the role of catalyst's intrinsic oxygen in plasma system was detected.

Additional Results

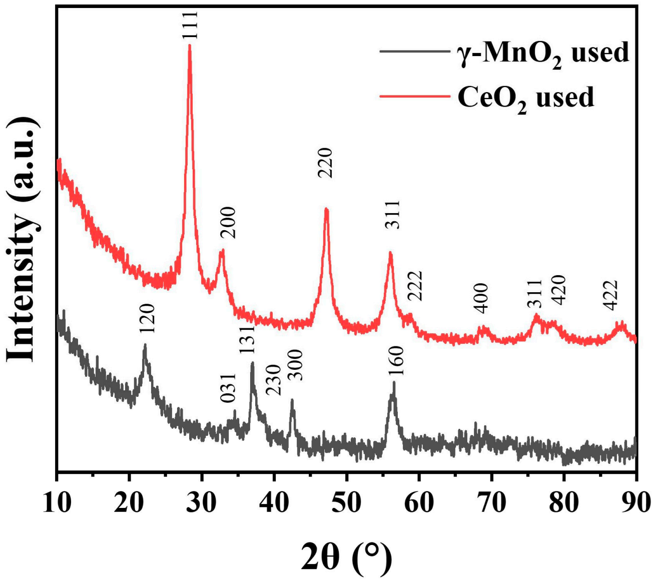

Figure S2. XRD pattern of used γ-MnO_2 and CeO_2.

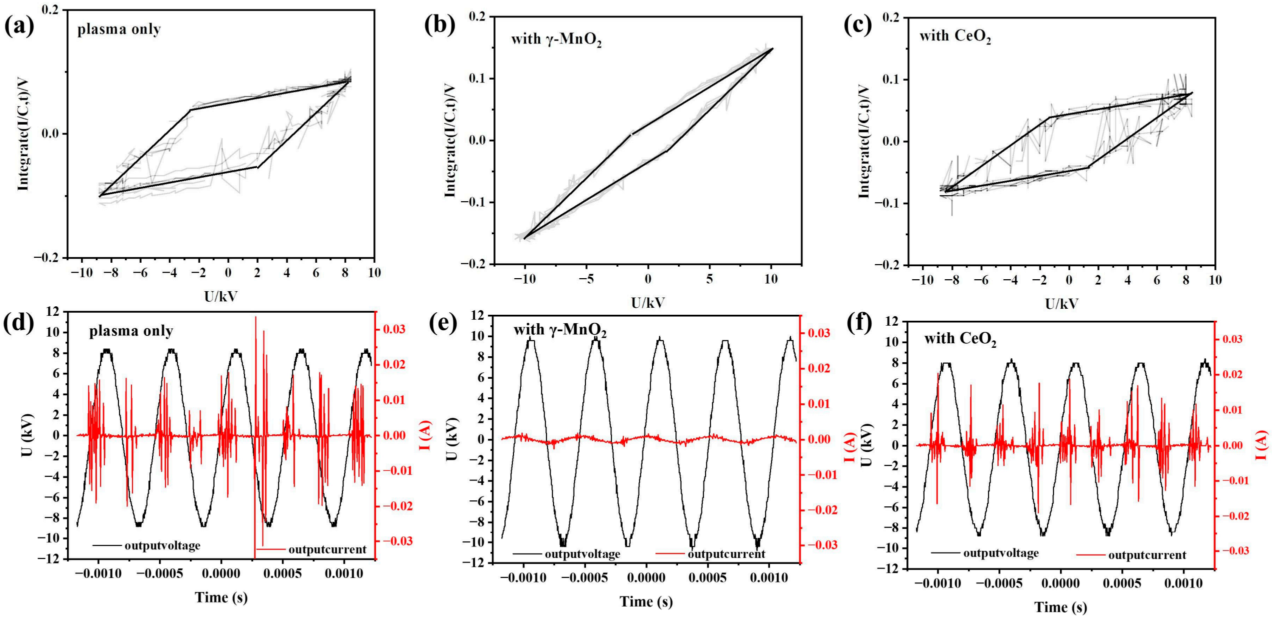

Figure S3. Lissajous curves for the (a) plasma-only system, plasma catalysis system with (b) γ-MnO_2, and (c) CeO_2; voltage and current waveforms for the (d) plasma-only system, plasma catalysis system with (e) γ-MnO_2 and (f) CeO_2 at an input power of 1 W.

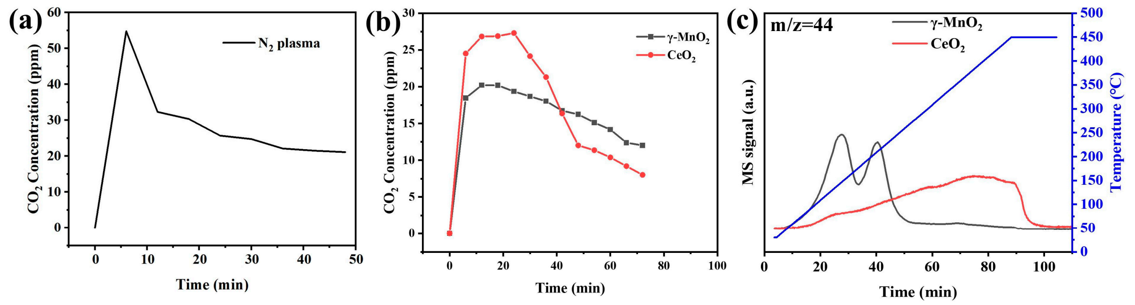

Figure S4. (a) CO_2 production in methanol plasma-catalytic experiment without catalysts (b) CO_2 production in catalytic experiments at room temperature without oxygen in the gas phase; (c) CO_2-TPD-MS profiles for catalysts after the catalytic experiments at room temperature (methanol concentration: 400 ppm, balance gas: N_2, gas flow rate: 100 mL·min[-1]).

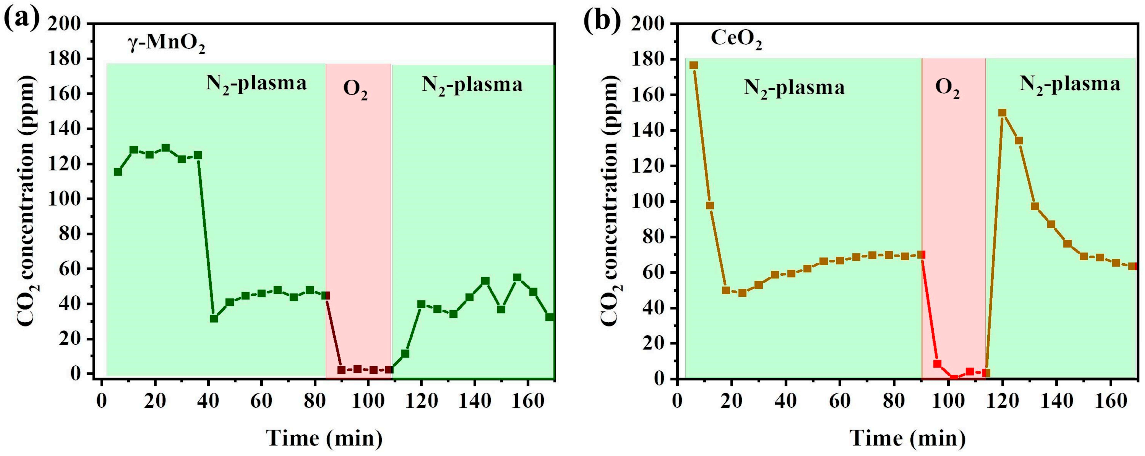

Figure S5. Evolution of outlet CO_2 of the plasma catalyst systems with different atmosphere (methanol concentration: 400 ppm, balance gas: N_2, catalyst amount: 0.1 g, gas flow rate: 100 mL·min[-1], temperature: 30°C, power: 1 W).

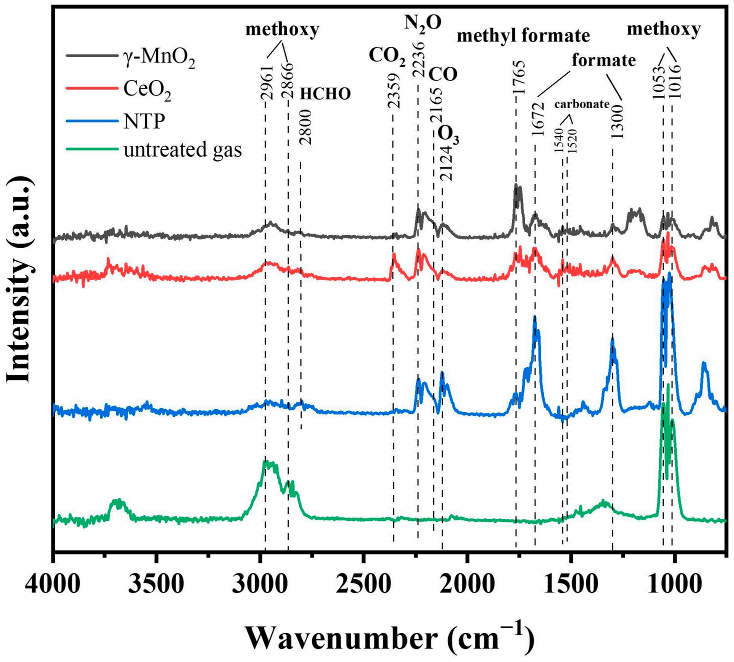

Figure S6. The FT-IR spectra of the plasma area during the reaction (methanol concentration: 400 ppm, balance gas: air, gas flow rate: 100 mL·min[-1], 1.9 kHz, 0.6 W).

Results of complementary *in situ* plasma FT-IR experiments

The first experimental results revealed the role of gas-phase oxygen in the surface reaction. After the removal of moisture and impurities by catalyst pretreatment, methanol/N_2 was introduced into the reactor. Figure S7 shows that a small quantity of carbonate species and CO_2 were formed on the surfaces of the two catalysts, demonstrating that the catalysts' intrinsic oxygen was involved in oxidation of methanol without gas-phase oxygen at room temperature. The catalytic process was as follows: the adsorbed methanol was oxidized to a small amount of carbonate, and then decomposed into a small amount of CO_2.

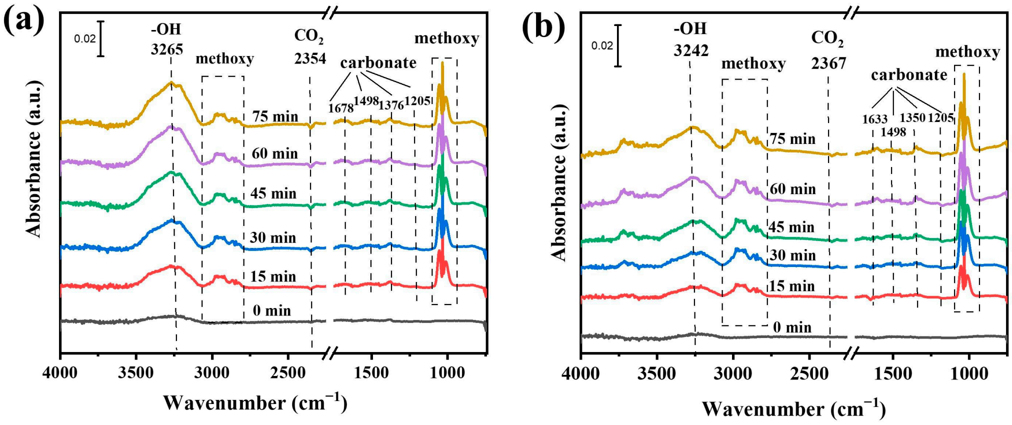

Figure S7. *In situ* FT-IR spectrum of methanol adsorption for (**a**) γ-MnO_2 and (**b**) CeO_2 at room temperature (methanol concentration: 400 ppm, balance gas: air, gas flow rate: 100 mL·min^{-1}).

Then the injecting gas was switched to high purity nitrogen, and after purging the methanol from the reactor, the plasma was turned on under the nitrogen atmosphere, and IR spectra were obtained, as shown in Figure S8. At this time the nitrogen plasma reacted with the intrinsic oxygen of catalysts, and some nitrogen-containing intermediate species were observed on the catalyst surface on both catalysts, and the IR peak of amide was observed at 1631 cm^{-1} and the peak of NO_3^- was at 1365 cm^{-1} [1,2]. The concentration of amide gradually decreased with time, while NO_3^- gradually increased., suggesting that there was a conversion of amide to NO_3^-. That is to say, some nitrogen-containing intermediate species were decomposed into simple nitrogenous salts.

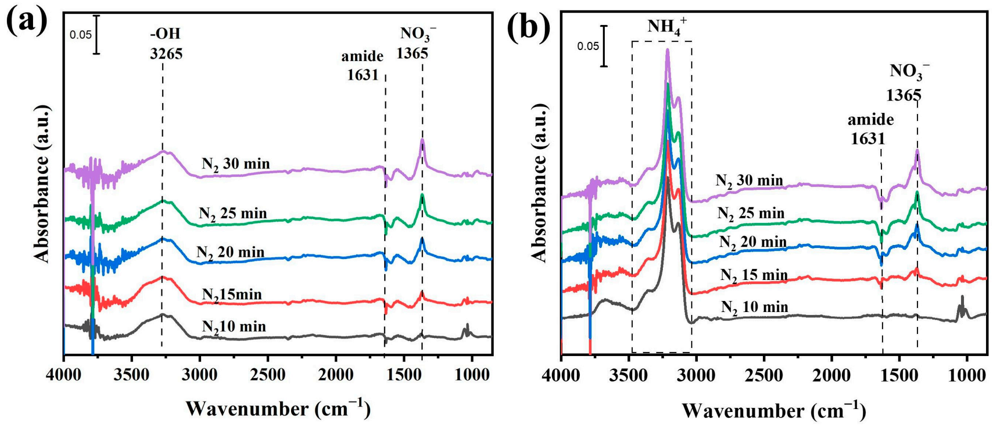

Figure S8. *In situ* FT-IR spectrum of N_2 plasma catalytic oxidation for (**a**) γ-MnO_2 and (**b**) CeO_2 after the adsorption at room temperature (nitrogen flow rate: 10 mL·min^{-1}, 1.9 kHz, 0.9 W).

Maintaining the plasma on, and then switching the gas from high purity nitrogen to dry air. As shown in Figure S9, a large number of formates were generated (the infrared peak of 1351 cm^{-1} is the symmetric vibration peak of formates) [3], indicating that oxygen in the gas phase, including excited oxygen species and ozone, was involved in oxidation of methanol on the surface of catalysts. Compared with the nitrogen-containing active species, the oxygen-active species in the gas phase were more active and oxidized methanol and its intermediates into formates.

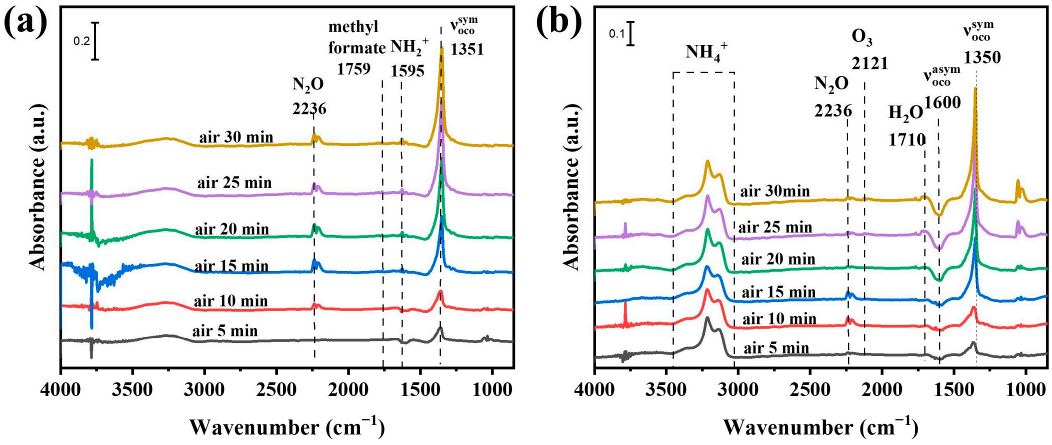

Figure S9. *In situ* FT-IR spectra of methanol degradation by catalyst own oxygen species combined with air plasma for (**a**) γ-MnO_2 and (**b**) CeO_2 (N_2 flow rate: 10 mL·min^{-1}, 1.9 kHz, 0.6 W).

The second experimental results revealed the role of the intrinsic oxygen of catalysts in the surface reaction. After pretreatment of the catalyst, a methanol/N_2 mixture was introduced into the *in situ* reactor, and after the catalyst was saturated with methanol, high-purity oxygen was introduced. Then the atmosphere switched to nitrogen to remove the residual oxygen in the reactor, and turn on the plasma, at which time there was no oxygen in the gas phase and the most source of oxygen was the intrinsic oxygen of catalysts. Figure S10 shows the IR spectra under nitrogen discharge conditions at this time, no O_3 peaks are observed on the surface of both catalysts, indicating that the intrinsic oxygen of catalysts was not excited by the nitrogen plasma to form ozone. Interestingly, the IR peak of formate (1350 cm^{-1}) was also observed when there is no oxygen in the gas phase, suggesting that the intrinsic oxygen of catalysts combined with the nitrogen plasma exhibited the ability to oxidize methanol. The hydroxyl group dissociated from methanol adsorbed on the catalyst surface was not observed at 3000–3500 cm^{-1}, suggesting that this step of dissociation requires the participation of gas-phase oxygen and that the intrinsic oxygen of catalysts combined with the nitrogen plasma is not sufficient to dissociate the hydroxyl group from methanol. In the absence of gas-phase oxygen, a large concentration of formate was detected on the surface of both catalysts, indicating that the oxidation of formate was the rate-limiting step.

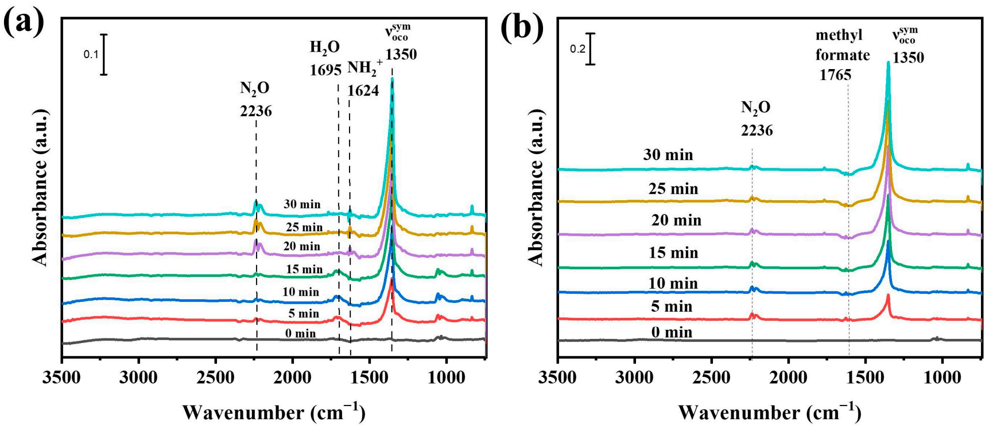

Figure S10. In situ FT-IR spectra of methanol degradation by catalyst own oxygen species combined with nitrogen plasma for (**a**) γ-MnO_2 and (**b**) CeO_2 (N_2 flow rate: 10 mL·min^{-1}, 1.9 kHz, 0.6 W).

References

[1] Chan-chan L H, González-garcía G, Vargas-coronado R F, et al. Characterization of model compounds and poly (amide-urea) urethanes based on amino acids by FTIR , NMR and other analytical techniques[J]. European Polymer Journal, 2017, 92: 27–39.

[2] Cunha M C P M, Weber M. On the adsorption and reduction of NO_3^- ions at Au and Pt electrodes studied by in situ FTIR spectroscopy[J]. Journal of Electroanalytical Chemistry, 1996, 414(2): 163–170.

[3] Lustemberg P G, Bosco M V., Bonivardi A, et al. Insights into the Nature of Formate Species in the Decomposition and Reaction of Methanol over Cerium Oxide Surfaces: A Combined Infrared Spectroscopy and Density Functional Theory Study[J]. Journal of Physical Chemistry C, 2015, 119(37): 21452–21464.
